# Large-scale analysis of human gene expression variability associates highly variable drug targets with lower drug effectiveness and safety

**DOI:** 10.1093/bioinformatics/btz023

**Published:** 2019-01-14

**Authors:** Eyal Simonovsky, Ronen Schuster, Esti Yeger-Lotem

**Affiliations:** 1 Department of Clinical Biochemistry & Pharmacology, Ben-Gurion University of the Negev, Beer-Sheva, Israel; 2 National Institute for Biotechnology in the Negev, Ben-Gurion University of the Negev, Beer-Sheva, Israel

## Abstract

**Motivation:**

The effectiveness of drugs tends to vary between patients. One of the well-known reasons for this phenomenon is genetic polymorphisms in drug target genes among patients. Here, we propose that differences in expression levels of drug target genes across individuals can also contribute to this phenomenon.

**Results:**

To explore this hypothesis, we analyzed the expression variability of protein-coding genes, and particularly drug target genes, across individuals. For this, we developed a novel variability measure, termed local coefficient of variation (LCV), which ranks the expression variability of each gene relative to genes with similar expression levels. Unlike commonly used methods, LCV neutralizes expression levels biases without imposing any distribution over the variation and is robust to data incompleteness. Application of LCV to RNA-sequencing profiles of 19 human tissues and to target genes of 1076 approved drugs revealed that drug target genes were significantly more variable than protein-coding genes. Analysis of 113 drugs with available effectiveness scores showed that drugs targeting highly variable genes tended to be less effective in the population. Furthermore, comparison of approved drugs to drugs that were withdrawn from the market showed that withdrawn drugs targeted significantly more variable genes than approved drugs. Last, upon analyzing gender differences we found that the variability of drug target genes was similar between men and women. Altogether, our results suggest that expression variability of drug target genes could contribute to the variable responsiveness and effectiveness of drugs, and is worth considering during drug treatment and development.

**Availability and implementation:**

LCV is available as a python script in GitHub (https://github.com/eyalsim/LCV).

**Supplementary information:**

Supplementary data are available at *Bioinformatics* online.

## 1 Introduction

**Fig. 1.**
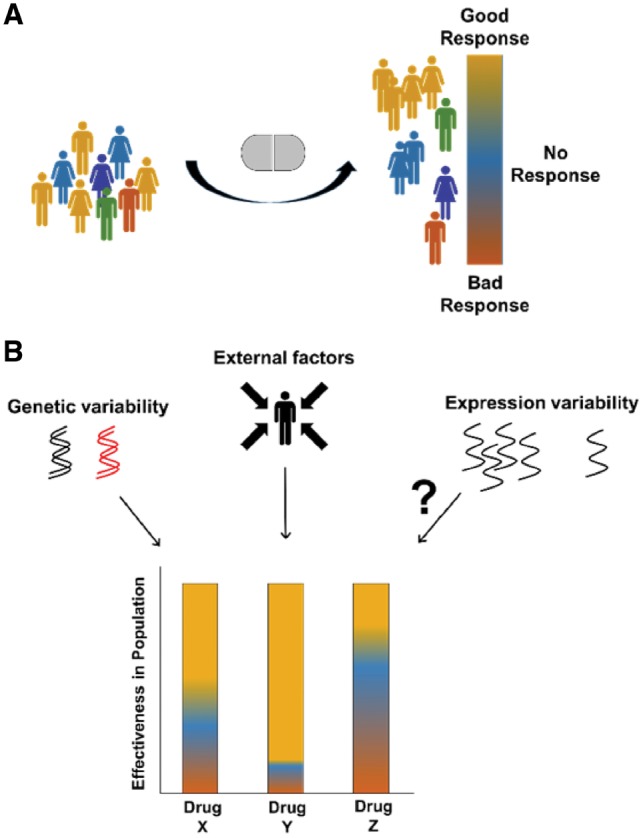
Drug effectiveness and causal factors. **(A)** Individuals showing similar symptoms may respond differently to the same drug treatment. A good response (orange) denotes an efficacious treatment with little adverse effects; No response (blue) denotes low treatment efficacy and little adverse effects; and bad response (red) denotes low treatment efficacy and severe adverse effects. **(B)** The effectiveness of drugs in the general population may be affected by genetic differences, such as genetic variation in pharmacogenes, and by external factors, such as patients’ physiological state, disease severity and environmental exposure. Here, we explore whether expression variability in drug targets might affect drug effectiveness as well (Color version of this figure is available at *Bioinformatics* online.)

The recognition that patients are a heterogeneous population is one of the pillars of the rising paradigm of precision medicine (Ashley, 2016). Due to this heterogeneity, patients that show a similar phenotype might respond differently to the same treatment regimen (Evans and Relling, 2004; Relling and Evans, 2015). Consequently, certain treatments might not be beneficial, or might even be harmful, to some patients (Schork, 2015) (Fig. 1A). The variability in the response to drugs may result from several factors including genetic polymorphisms or mutations, differences in the physiological state of patients, disease severity, and other external factors (Eichler *et al.*, 2011; Evans and McLeod, 2003).

A key factor known to determine the heterogeneous responsiveness among patients is genetic polymorphisms in pharmacogenes; genes that are targeted by the drug, or involved in drug absorption, distribution, metabolism and exertion (ADME) (Relling and Evans, 2015; Scharfe *et al.*, 2017). Significant efforts are invested in identifying variants of these genes (Bush *et al.*, 2016) and constructing guidelines for their sequencing in patients, in order to optimize therapy (Caudle *et al.*, 2014). The importance of these efforts is demonstrated by the variable responsiveness of patients to codeine, a commonly prescribed opiate indicated for the relief of pain and cough. Upon ingestion, codeine is converted by the hepatic enzyme Cytochrome P450 2D6 (CYP2D6) to several metabolites, including morphine. Genetic polymorphisms in CYP2D6 that are particularly frequent among Arabs and northeast Africans (10–30%) were associated with ultra-rapid metabolism of codeine (McLellan *et al.*, 1997). This ultra-rapid metabolism leads to increased levels of morphine, which may cause severe adverse effects up to respiratory repression (Crews *et al.*, 2014).

Heterogeneous responses to a drug might also arise due to variable expression of its pharmacogenes (Fig. 1B). For example, mRNA levels of the human anion transporter gene HOAT3 were reported to correlate with renal excretion of the anionic drug cefazolin (Sakurai *et al.*, 2004). The variable expression of genes, which stems from the stochasticity of the expression process, was associated with genetic attributes, such as histones modifications, and with functional attributes, such as their related cellular processes (Alemu *et al.*, 2014; Newman *et al.*, 2006). House-keeping genes, for example, were shown to be less variable than stress response genes, demonstrating the beneficial aspects of both low and high expression variability (Newman *et al.*, 2006 and reviewed by Raj and van Oudenaarden, 2008). Here, we hypothesize that drugs with variably expressed targets are more likely to elicit variable responses among patients than drugs with uniformly expressed targets. The variable expression of drug target genes could stem not only from polymorphisms within these genes, but also from differences in lifestyle (Ornish *et al.*, 2008), environmental exposures (Jung and Sang, 2007), or sequence variation in remote genomic regions as in eQTLs (Consortium *et al.*, 2017). Importantly, the availability of hundreds of RNA-sequencing profiles of human tissues, enabled by the GTEX project, allows one to measure gene expression variability at unprecedentedly broad scale (Consortium *et al.*, 2017).

The study of gene expression variability was carried mostly in model organisms at the cellular level (reviewed in (Chalancon *et al.*, 2012; Raj and van Oudenaarden, 2008). The classic variability measure, SD, was shown to be biased towards genes with high expression levels, and was replaced by the coefficient of variation (CV). CV, which is computed as SD divided by the mean, normalized SD and produced a unit-less measure. Later on, CV was shown to be biased toward genes with low expression levels (Alemu *et al.*, 2014; Silander *et al.*, 2012). Ensuing measures estimated the variability of a gene in the context of other genes (Newman *et al.*, 2006; Silander *et al.*, 2012), e.g. as a distance between a gene’s variability and the variability of genes with similar expression levels (denoted Distance from the Median CV, DM for short, Newman *et al.*, 2006). A relatively recent measure, termed expression variation (EV), assumed that expression variability is gamma-distributed across expression levels, and computed the variability of a gene as the ratio between its observed and expected gamma-distributed variability (Alemu *et al.*, 2014).

Several studies analyzed the expression variability of human genes. Alemu *et al.* (2014), who developed the EV measure, collected microarray-based gene expression profiles from the gene expression omnibus. They showed that EV scores were associated with several genetic and functional characteristics, e.g. variable genes were shown to function in extra-cellular pathways and to be involved in human diseases. Another study used median absolute deviation (MAD) to estimate the variation of single-cell and bulk RNA-sequencing measurement of gene expression (Wu *et al.*, 2014). A different study used linear models to analyze gene expression variability across organs and species to investigate whether organ-specific transcriptional patterns dominate over species-specific patterns, or vice versa (Breschi *et al.*, 2016). This study found a continuum in the spectrum of gene expression variability, ranging from genes with tissue-dominated variability, which was more likely associated with diseases, to genes with species-dominated variation, which reflected evolutionary distance. A drug-related study analyzed the expression variability of ADME genes in human liver by using SD and CV (Yang *et al.*, 2013). It showed that ADME genes tend to be more variable than other protein-coding genes, and suggested that this could lead to variable responsiveness. A subsequent study used RNA-sequenced expression profiles of four tissues to analyze the expression levels and alternative splicing of 389 genes with key roles in drug disposition (Chhibber *et al.*, 2017). It identified substantial variability in gene expression particularly among drug transporters and drug metabolizing enzymes. RNA-sequencing data of around 30 human tissues was used recently to analyze the tissue-specificity of drug targets (Uhlen *et al.*, 2015). Drug target genes were shown to be enriched for tissue-elevated proteins, and 30% of all drug targets were found to be expressed in all tissues. However, less is known about the expression variability of drug target genes across tissues.

Here, we harness RNA-sequenced expression profiles of 19 human tissues that were collected in a uniform way by the GTEx consortium (Consortium *et al.*, 2017) to test whether expression variability could be linked with drug effectiveness. We introduce a novel, general measure for expression variability, termed local coefficient of variation (LCV), which is highly correlated with DM and EV yet is more robust and does not assume a predefined variability distribution. We found that drug target genes were significantly more variable than other protein-coding genes. Upon analyzing 113 approved drugs for which feedbacks from patients and physicians were available, we found that drugs that target highly variable genes tend to be less effective. Furthermore, upon analyzing 1033 approved drugs and 86 drugs that were withdrawn from the market; we found that withdrawn drugs tend to target more variable genes than approved drugs. Similar results were obtained upon analyzing separately data from men and women. These results suggest that expression variability of drug target genes is a contributing factor to the variability in drug responses among patients.

## 2 Materials and methods

### 2.1 Development of the LCV algorithm

**Fig. 2.**
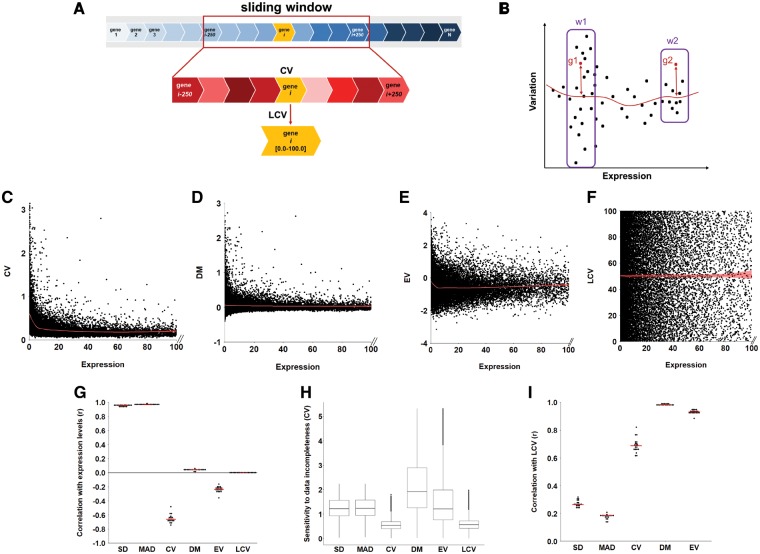
Comparison between LCV and other expression variability measures. **(A)** The scheme for calculating LCV per gene per tissue. The top row shows genes ordered by their median expression levels, scanned by using a sliding window centered at gene g_i_. The middle row shows genes in that window, with color intensity reflecting the percentile of their CV value relative to each other. The LCV of g_i_ is set to the percentile of its CV value amongst these genes. The sliding window then slides to g_i+1_ and the processes repeats. **(B)** Toy example of the intuition behind the LCV measure. g1 and g2, are similarly variable genes. A direct measure of the distance or the ratio between gene variability and a variability expression-dependent function (red line) will score both genes with similar variable values. LCV will score g2 as most variable in window w2 (LCV of 100) and g1 as highly variable in window w1, yet less variable than g2. **(C)** The CV scores of 21 733 genes expressed in the cerebellum are plotted against their median expression level. The local regression fitting line, depicted in red, shows that CV is increased amongst lowly expressed genes (*r* = −0.74, *P* < 1*10^−15^, Spearman correlation). **(D)** The DM scores of 21 733 genes expressed in the cerebellum are plotted against their median expression level. The local regression fitting line, depicted in red, is unbiased toward lowly expressed genes (*r* = 0.040, *P* = 6*10^−10^, Spearman correlation). **(E)** The EV scores of 21 733 genes expressed in the cerebellum are plotted against their median expression level. The local regression fitting line, depicted in red, shows that EV is increased amongst lowly expressed genes (*r* = −0.24, *P* < 1*10^−15^, Spearman correlation). **(F)** The LCV scores of 21 733 genes expressed in the cerebellum are plotted against their median expression level. The local regression fitting line, depicted in red, is unbiased toward lowly expressed genes (*r* = 0.0013, *P* = 0.8, Spearman correlation). **(G)** The expression biases observed for different variability measures in 19 tissues. Each dot represents the Spearman correlation coefficient between gene expression variability and gene expression level in a specific tissue. Red horizontal lines show the median correlation coefficient. LCV and DM are unbiased by expression levels. Other variability measures range between mild (EV) to moderate (CV) to high (SD and MAD) absolute correlation with expression levels. **(H)** The sensitivity to incompleteness of data observed for different variability measures. Gene variability scores per gene per tissue were computed for the same five data subsets. Results per measure are represented in each boxplot. LCV and CV scores were the most robust towards data incompleteness (CV of 0.54 and 0.57, respectively). SD, MAD, DM and EV were more sensitive to data incompleteness (CV of 1.22, 1.23, 2.42 and 1.36, respectively). **(I)** The correlation between LCV scores and scores obtained via other variability measures in 19 tissues. Each dot represents the Spearman correlation coefficient obtained for a specific tissue. Red horizontal lines show the median correlation coefficient. LCV is highly correlated with DM and EV in all tissues. LCV is correlated to a lesser extent with CV and is only mildly correlated with MAD and SD

We aimed to develop an unbiased and general method for estimating the expression variability of a gene. Our proposed method, LCV, converts CV, the consensus measure for variability, to a ranking approach that compares between genes with similar expression levels. By that, it avoids the expression bias observed for CV (Silander *et al.*, 2012), and can be easily compared across different genes and datasets (Fig. 2A). To implement LCV, we first sort all genes per tissue according to their median expression level across all samples in the dataset, and compute their CV. To obtain a local CV measure, we use a sliding window that is centered at gene g_*i*_, and rank the CV of g_*i*_ relative to the CV of other genes located in that window. We then set the local CV of g_*i*_, denoted LCV(g_*i*_), to the percentile that fits the ranking of its CV. Henceforth, genes with LCV close to zero were considered invariable, while genes with LCV close to 100 were most variable. By sliding the window across all genes we compute LCV for all genes one by one. Several other methods compute a local variability measure that assesses the variability of a gene relative to the variability of genes with similar expression levels, by using distance (Newman *et al.*, 2006; Silander *et al.*, 2012) or ratio-based functions (Alemu *et al.*, 2014). The advantage of a ranking-based approach is demonstrated by the toy example in Figure 2B. Any distance or ratio-based function will score genes g1 and g2 similarly. However, within the context of similarly expressed genes (windows w1 and w2), it is clear that g2 is more variable than g1. By using ranking, LCV will score g2 as most variable (LCV of 100) and g1 as less variable. This refinement is meaningful especially when comparing between genes with highly distinct expression levels (Fig. 2B). We applied LCV to the rich dataset of human tissue expression profiles measured via RNA-sequencing by the GTEx consortium (Consortium *et al.*, 2017). We focused on the 19 tissues with at least 10 available profiles each (Supplementary Fig. S1), and computed LCV for window sizes ranging between 10 and 10 000 genes. Windows were generally centered at each gene except for the two ends of the scale, where they were shifted to include *m* adjacent genes. LCV values computed for window sizes between 100 and 1000 genes were similar to each other as shown by their Spearman correlations (*r* > 0.99, Supplementary Fig. S2), and remained quite similar even when extremely small (10 genes) or large (10 000 genes) window sizes were used (*r* > 0.90, Supplementary Fig. S2), supporting the robustness of the LCV method. The distributions of the variability calculated for cerebellum profiles using different window sizes appear in Supplementary Figure S3.

### 2.2 Evaluating the expression bias and robustness of LCV and other expression variability measures

We tested LCV and other expression variability measures, including SD, MAD, CV, DM and EV, for expression level biases by applying them to the human tissue expression profiles described above, and computing the Spearman correlation between variability and expression. As demonstrated with cerebellum profiles, CV values were negatively correlated with gene expression levels and were significantly biased toward lowly expressed genes (*r* = −0.74, *P* < 1*10^−15^, Fig. 2C). Other local variability assessment measures, DM and EV, showed none or moderate bias toward lowly expressed genes (500-gene window, *r* = 0.040, *P* = 6*10^−10^ and *r* = −0.24, *P* = 1*10^−15^, respectively, Fig. 2D and E). LCV was the least biased measure (500-gene window, *r* = −0.0013, *P* = 0.8, Fig. 2F). Similar results were obtained upon applying the measures to all 19 tissues in our dataset (Fig. 2G): SD, MAD and CV were consistently correlated with, and thus biased by, expression levels (median *r* of 0.95, 0.97 and −0.66, respectively). EV was mildly correlated with expression levels (median *r* = −0.24), while DM and LCV were unbiased (median *r* of 0.04 and 3.5*10^−4^, respectively). Even when computed for window sizes ranging between 10 and 1000 genes, LCV values did not correlate with expression levels (|*r*| < 3.5*10^−4^, Supplementary Figs S3 and S4). Only upon applying extremely large window size (10 000 genes), LCV values correlated mildly with expression levels (median *r* = −0.19, Supplementary Fig. S4), similarly to the correlation obtained for EV.

Next, we tested the robustness of the different measures to data incompleteness (Fig. 2H). We created five subsets of randomly selected samples, containing 50% of the available samples per tissue. For each measure, we calculated gene expression variability in each subset, resulting in five variability scores per gene per tissue. Then, we estimated the variation in the variability scores across the five subsets per gene and tissue. We estimated the variation by using CV (SD divided by the mean) since it is a straightforward unit-less measure, which allowed us to compare between the variations observed per measure. Thus, low CV values imply that the variability estimates are relatively uniform, while high CV values imply high sensitivity (low robustness) to data incompleteness. We then grouped together the CV scores of all genes across all tissues. This was repeated for each measure by using the same subsets of samples. The variation per measure appears in Figure 2H. Notably, CV and LCV had the lowest variations (median CV of 0.54 and 0.57, respectively), while SD, MAD, EV and DM were more variable (median CV of 1.22, 1.23, 1.36 and 2.42, respectively). Similar results were obtained upon repeating the analysis separately per tissue. Thus, LCV is more robust to data incompleteness than other measures that use local assessment of variability.

To further validate LCV, we computed the Spearman correlations between LCV and each of the other expression variability measures (Fig. 2I). As might be expected, LCV had relatively low correlations with SD and MAD (median *r* = 0.26 and 0.18), was better correlated with CV (median *r* = 0.69), and was highly correlated with the DM and EV (median *r* = 0.98 and 0.93, respectively). The high correlation between EV and LCV supports the validity of LCV, and shows that a variability measure can remain unbiased without assuming a predefined variability distribution.

### 2.3 Implementation details

LCV was implemented in Python, and is available in GitHub (https://github.com/eyalsim/LCV). When window size was not explicitly mentioned, a 500-gene window was used. SD and CV (computed as SD divided by the mean) were computed straightforwardly. MAD was computed as the median of the absolute deviations from the median of a gene’s expression level in different samples. DM was computed as the distance from the median CV in a 500-gene window. R code for the EV function (Alemu *et al.*, 2014) was downloaded as part of the antiProfiles package (Corrada Bravo *et al.*, 2012). In the locfit function, nearest neighbor component of the smoothing parameter was set to nn = 0.3. Other parameters were set to their default values.

#### 2.3.1 Human gene expression data

Tissue expression profiles of normal human samples were obtained from GTEx portal on February 22, 2017 (version 6p) (The GTEx Consortium, 2015). In general, GTEx did not include diseased samples, and we used only samples that were denoted with traumatic injury as the cause of death, in order to further increase their reliability as proxy for healthy tissues. We analyzed tissues for which 10 or more samples were available, which amounted to a total of 296 samples across 19 tissues (Supplementary Fig. S1). Raw read counts were normalized to obtain the same library size for every sample by using the trimmed mean of M-values (TMM) method by the edgeR package (Robinson *et al.*, 2010), as described elsewhere (Basha *et al.*, 2017). Genes with at most 10 raw counts in all samples were removed before normalization, as these genes were typically regarded as noise. In the comparison between LCV and other variability measures (Fig. 2), expression variability was computed for all genes expressed above 0 counts per million (cpm) in at least 80% of the samples of a tissue. This cutoff was used in order to allow for lowly expressed genes that were measured in a large enough number of tissue samples that enables variability estimation. This resulted in a dataset of 29 421 genes with available variability scores (Supplementary Table S1).

#### 2.3.2 Expression variability of protein-coding and essential genes

**Fig. 3.**
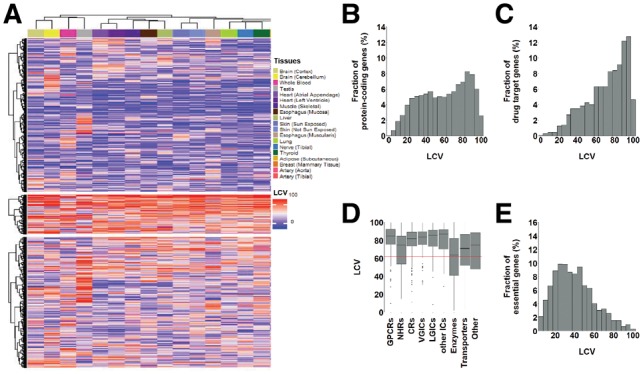
Gene expression variability across human tissues is higher among drug-target genes. **(A)** Hierarchical clustering of 5078 globally expressed genes by their LCV values reveals common regulatory patterns between tissues. Three main gene clusters were identified. Physiologically related tissues have similar colors and tend to be co-clustered. Gray bars show the median LCV of each gene. **(B)** Histogram of median LCV scores across tissues for 14 659 protein-coding genes. Protein-coding genes show a slight bi-modal distribution. **(C)** Histogram of median LCV scores across tissues for 1176 drug target protein-coding genes. In contrast to the distribution of protein-coding genes, drug target genes tend to be highly variable. **(D)** The expression variability of drug target genes shown per gene family, relative to the median LCV score of protein-coding genes (red line). All drug target gene families, except enzymes, tend to be more variable than protein-coding genes. Gene families include G protein–coupled receptors (GPCR), nuclear hormone receptors (NHR), catalytic receptors (CR), voltage-gated ion channels (VGIC), ligand-gated ion channels (LGIC), other ion channels (other IC), transporters and other. **(E)** The expression variability of the 1406 essential genes shows that they tend to be less variable that protein-coding genes

Protein-coding genes were downloaded from Ensembl Biomart (Yates *et al.*, 2016). Expression variability was computed for all genes that were expressed above 7.0 cpm in at least 80% of the samples of a single tissue, and thus reliably measured in a large enough number of tissue samples. This resulted in 14 659 protein-coding genes with available variability scores (Supplementary Table S2). The heatmap (Fig. 3A) included genes with available LCV values in all 19 tissues, and was computed by using R’s ComplexHeatmap package (Gu *et al.*, 2016). Genes were clustered based on Euclidean distance matrix and ward.D2 clustering method. Tissues were clustered based on spearman distance matrix and ward.D2 clustering method. Gene ontology (GO) term enrichment analysis was performed via Gorilla (Eden *et al.*, 2009). GO enrichment per tissue was computed for a gene list ranked by LCV values. GO enrichment per cluster was computed relative to a background list comprising all genes considered in the heatmap analysis. Data for gene essentiality were taken from a screen that measured gene essentiality in four cell lines and assigned negative scores to essential genes (Wang *et al.*, 2015). To simplify the interpretation, we negated the essentiality values, so that essential genes would have positive scores. We compared between gene essentiality and LCV by using, per gene, its median LCV value across tissues and its median essentiality value across cell lines. Essential genes were defined as genes whose negative median impact on growth was at the top 10%.

#### 2.3.3 Data of drugs and drug-related genes

Drug target and ADME genes associated with approved and withdrawn drugs were downloaded from DrugBank (Wishart *et al.*, 2006). Drug-target proteins were defined according to DrugBank, as a ‘protein, macromolecule, nucleic acid or small molecule, to which a given drug binds, resulting in an alteration of the normal function of the bound molecule’ (https://www.drugbank.ca/documentation). Drug-target gene families were downloaded from PharmGKB (Whirl-Carrillo *et al.*, 2012).

Relative efficacy (RE) scores of drug-disease pairs were downloaded from Guney *et al.* (2016). There, to compute RE scores, text-mining techniques were applied to reports that were submitted to the Adverse Event Reporting System (FAERS) operated by the FDA (https://open.fda.gov/data/faers/). Based on these reports, Guney *et al.* computed the RE score for each drug-disease pair, as the number of reports stating that the drug was ineffective for treating the disease ($n_{\text{ineffective}})$, divided by the number of reports stating the most common complaint, which could be that the drug was ineffective ($n_{\text{most}\;\text{common}\;\text{complaint}}$, Equation 1).

$${\text{RE}}_{\text{drug}-\text{disease}\;}=1\;\frac{n_{\text{ineffective}}}{n_{\text{most}\;\text{common}\;\text{complaint}}}(1)$$

Thus, if a drug was mostly ineffective for treating the disease, RE score was close to 0; if a drug had almost none ineffective reports, RE was close to 1. Notably, RE was computed only for drug-disease pairs with at least 10 reports.

The association between drugs and the anatomical systems that they were intended for was based on the anatomical therapeutic chemical classification system (ATC codes) of the World Health Organization. We used the first level of the hierarchical ATC classification, which specifies a total of 14 anatomical systems. The association between a drug and its ATC code(s) was downloaded from DrugBank (Wishart *et al.*, 2006).

#### 2.3.4 Gender analysis

Expression variability was computed separately for expression profiles of samples from men or women. Analysis did not include genes located on the X or Y chromosomes, and was carried in the subset of tissues with at least five profiles of samples from male and female subjects. A total of 983 drugs had target genes in our dataset for males, 1001 drugs had targets genes in our dataset for females, with 981 of them common to both.

## 3 Results

### 3.1 Expression variability of protein-coding genes across tissues

We started by analyzing the expression variability of protein-coding genes in each of the 19 human tissues in our dataset (available as Supplementary Table S2). First, we tested whether genes with low or high variability were enriched for certain GO terms. In agreement with previous reports (Alemu *et al.*, 2014), genes with low variability tended to be nuclear (median enrichment across tissues *P* = 2.47*10^−10^), while highly variable genes tended to be extracellular (median enrichment across tissues *P* = 3.20*10^−33^), fitting with the previously observed tendency of signaling-related genes to be variable (Newman *et al.*, 2006). Next, we compared the expression variability of genes across tissues. For this, we hierarchically clustered the subset of 5078 globally expressed genes according to their LCV in the 19 tissues (Fig. 3A). Physiologically related tissues clustered together, for example brain cortex with cerebellum, left ventricle of the heart with atrial appendage of the heart, and breast with adipose, revealing common expression variability patterns that appear to reflect common regulatory programs (Fig. 3A). The largest gene cluster included 2413 genes that were mostly invariable across tissues. These genes were enriched for nuclear genes (*P* = 1.07*10^−16^) and for nucleic acid metabolic processes (*P* = 4.69*10^−14^). A second cluster of 613 genes included mostly variable genes, and was mainly enriched for cellular response pathways, such as response to chemicals (*P* = 2.23*10^−8^). A third cluster included 2052 genes with mixed variability across tissues, and was enriched for extracellular organelle (*P* = 9.21*10^−9^), exosome (*P* = 3.06*10^−9^) and membrane (*P* = 3.27*10^−9^). These features were consistent with known features of variable and invariable genes (Alemu *et al.*, 2014).

### 3.2 Drug target genes tend to be more variable than protein-coding genes

Our next step was to analyze the expression variability of drug target genes. We gathered data of 1076 FDA-approved drugs and their 1176 targets from DrugBank (Wishart *et al.*, 2006). We first asked whether protein-coding drug target genes had similar expression variability as protein-coding genes. For this, we associated each gene with its median LCV across tissues, and compared the LCV distributions of protein-coding (Fig. 3B) and drug target genes (Fig. 3C). Protein-coding genes had a slight bi-modal distribution, including a small peak around LCV of 45 (5.66% of genes, relative to a plateau of 5.01% in its adjacent regions), and a more distinguishable peak at 85 (8.06% of genes, relative to 6.48% in its adjacent regions). To better understand which genes contribute to each peak, we created a group of genes with LCV scores around 45 (35–55), and a group of genes with LCV scores around 85 (75–95), and computed their enrichment for GO terms relative to all protein-coding genes. The group of genes with LCV scores around 45 was only slightly enriched, in agreement with being close to the median LCV and quite similar to its neighboring regions. One of the few enriched terms was intracellular genes (*P* = 2.64*10^−24^), including mitochondrial and nuclear genes (*P* = 5.20*10^−16^ and 5.21*10^−14^, respectively), similarly to genes with low LCV. In contrast, the group of genes with LCV scores around 85 was highly enriched for multiple terms, many of them related to extracellular and signaling processes, functions or cellular components. Highly enriched terms included cellular adhesion (9.02*10^−30^), developmental process (4.75*10^−29^), signaling process (*P* = 3.16*10^−16^), signaling receptor activity (3.97*10^−40^), intrinsic component of membrane (*P* = 1.27*10^−71^), extracellular genes (*P* = 3.37*10^−41^) and plasma membrane (*P* = 3.95*10^−71^), as well as differentiation processes (*P* = 3.06*10^−17^).

Unlike all protein-coding genes, the subset of drug target genes had a distinct unimodal distribution that peaked at LCV of 95, and were significantly more variable (*P* = 1.1*10^−50^, Mann-Whitney test). The high variability of drug target genes was not dominated by a specific gene family, but rather was common to several families (Fig. 3D). This high variability was also observed upon applying EV to these data (*P* = 3.3*10^−43^, Mann-Whitney test), suggesting that drug target genes are more prone to elicit variable responses than other protein-coding genes.

We also analyzed expression variability of essential genes (Fig. 3E). For this, we used a screen that measured the essentiality of roughly 18 000 human genes, 14 056 of which were in our dataset (Wang *et al.*, 2015). In accordance with Wang *et al*., we referred to essential genes as the top 10% of the genes whose manipulation leads to reduced growth (see Section 2). These genes were less variable than protein-coding genes, with a unimodal distribution that peaked at LCV of 25. Looking at the entire set of genes, there was a negative correlation between expression variability and essentiality: Essential genes tended to be less variable, suggesting that they are under strict regulatory programs, while less essential genes were more likely variable (*r* = −0.25, *P* = 1.5*10^−200^ Spearman correlation; Supplementary Fig. S5).

### 3.3 Variability of drug targets is linked to drug effectiveness

**Fig. 4.**
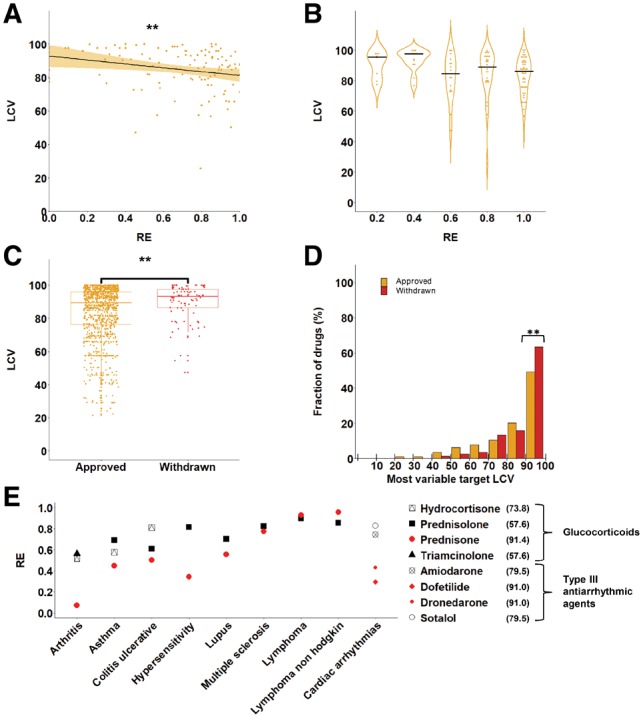
Expression variability of drug targets is linked to drug effectiveness. **(A)** The RE of 113 drugs is plotted against the expression variability (LCV) of their most variable target gene, showing a mild but significant negative correlation between RE and LCV (*r* = −0.29, *P* = 0.0018, Spearman correlation). **(B)** Analysis of 113 drugs shows that the LCV of drug target genes for drugs with similar RE values shows that less effective drugs (RE below 0.4) tend to have highly variable targets (LCV > 90). **(C)** The expression variability of drug target genes is shown for the disjoint sets of 1033 approved drugs and 86 drugs that were withdrawn from the market. Each dot represents the most variable target of the drug. The targets of withdrawn drugs are significantly more variable than the targets of approved drugs (*P* < 0.005, Mann-Whitney U test). **(D)** The distribution of 1033 approved drugs (gold) and 86 withdrawn drugs (red) according to the LCV of their most variable target shows that 64% of the withdrawn drugs have highly variable genes (LCV > 90) relative to 49% of the approved drugs. ‘**’ denotes hypergeometric *P* = 0.0097. **(E)** The RE of eight different drugs, including four glucocorticoids indicated for eight diseases, and four Type III antiarrhythmic agents indicated for cardiac arrhythmias. Each symbol represents a different drug and is colored in red if a target of the drug has a highly variable expression (LCV > 90, appearing in parenthesis per drug). In seven of the nine diseases the drugs with the highly variable targets have the lowest efficacy (Color version of this figure is available at *Bioinformatics* online.)

Given the high variability of drug target genes, we went on to test whether it could be linked with the variable responsiveness to drugs in the general population. For this, we first used a dataset of the RE of drugs, computed based on text-mining of feedback reports made by diseased patients and physicians to FAERS (Guney *et al.*, 2016). RE scores ranged between zero, for ineffective drugs, and one, for effective drugs. RE scores were computed for drugs indicated for well-studied, complex diseases and were available for 113 drugs with targets in our dataset. To test for a relationship between RE and expression variability we associated each drug with its most variable target. For drugs with multiple indications we used their median RE. We observed a mild but significant negative correlation between the RE of a drug and the LCV of its target (*r* = −0.29, *P* = 1.8*10^−3^, Spearman correlation, Fig. 4A). In particular, drugs with low RE (below 0.4) tended to target highly variable genes (median LCV = 96), whereas drugs with higher RE (above 0.4) tended to target less variable genes (median LCV = 86.3, Fig. 4B). This was also observed when using EV as the expression variability measure (*r* = −0.29, *P* = 2.1*10^−3^), supporting this observation.

To explore individual disease segments, we turned to the anatomical therapeutic chemical classification system (ATC codes) of drugs, made available by the World Health Organization. The first level of the hierarchical ATC classification specifies a total of 14 anatomical systems that drugs are intended for. For example, the antiarrhythmic drug, dofetilide, was classified to the cardiovascular system (Group C). We therefore grouped the drugs in our dataset by their ATC codes, and repeated the tests for each group of drugs. We first tested the correlation between LCV and RE (Supplementary Table S1). For this, we computed the correlation for groups consisting of at least 10 observations (i.e. drugs with available LCV and RE scores), resulting in 6 groups. In 2/6 groups (Group A: Alimentary tract and metabolism and group M: Musculo-skeletal system), LCV and RE were negatively and significantly correlated (*r* = −0.57 and −0.72, respectively, *P* < 0.05), consistently with the general trend. In the remaining groups, the correlation was insignificant, but mostly negative (Supplementary Table S1). We also tested the difference between approved and withdrawn drugs, which showed similar but weaker trends (Supplementary Table S2). To conclude, the small size of available data limited our ability to test each disease segment reliably. Nevertheless, in all cases where the result was significant, it was consistent with the general trend.

To further test whether drugs with highly variable targets were more prone to elicit negative responses in the population, we turned to examine approved and withdrawn drugs. We extracted from DrugBank 1033 FDA-approved drugs and 86 drugs that were withdrawn from at least one market, which had targets in our dataset. We then compared the expression variability of the most variable targets between the two classes of drugs (Fig. 4C). Targets of the withdrawn drugs were significantly more variable and differently distributed than targets of approved drugs (median LCV = 93.15 and 89.30, respectively, *P* = 0.005, Mann-Whitney test; *P* = 0.021, Kolmogorov–Smirnov-test). For example, 64% of the withdrawn drugs had targets with LCV above 90, relative to 49% of the approved drugs (hypergeometric *P* = 0.0097, Fig. 4D).

The negative effect that may be associated with highly variable targets is demonstrated by the family of glucocorticoids drugs, a subclass of steroid hormones commonly used for inflammatory diseases but also indicated for other usages. Although all glucocorticoids target the glucocorticoid receptor gene, NR3C1 (LCV = 57.6), hydrocortisone also targets the gene ANXA1 (LCV = 73.8), and prednisone also targets the highly variable HSD11B1 gene (LCV = 91.4). The population-wide effectiveness of these glucocorticoids was assessed for the anti-inflammatory conditions arthritis, asthma, colitis, hypersensitivity and lupus, as well as lymphoma, non-Hodgkin lymphoma and multiple sclerosis (Guney *et al.*, 2016). For six out of these eight diseases prednisone, which targets the highly variable HSD11B1 gene, was less effective than any of the other glucocorticoids (Fig. 4E). The only exceptions were lymphoma and non-Hodgkin lymphoma cancers, where regulatory patterns might be altered. Another example is presented by the family of Type III antiarrhythmic agents that is used for treating cardiac arrhythmias. RE values were available for four of these drugs, including dofetilide, dronedarone, amiodarone and sotalol (Guney *et al.*, 2016). Each of the four drugs targets between 3 to 18 genes, yet while the most variable target of amiodarone and sotalol was the gene KCNH2 (LCV = 79.5), the most variable target of dofetilide and dronedarone was the highly variable gene KCNK2 (LCV = 91.0). Dofetilide and dronedarone were less effective than amiodarone and sotalol in the general population, in accordance with the higher variability of their target (Fig. 4E). Table 1 presents the 10 most variable drug target genes and the respective drugs. LCV of all analyzed drug target genes and the respective drugs are available as Supplementary Table S3.

**Table 1.** The 10 most variable drug target genes and their respective drugs.

Target	LCV	No. of drugs	Drugs
SULT1E1	100	1	Cyclizine
HLA-DQB1	100	1	Insulin pork
SLC6A4	100	47	Fluvoxamine, amphetamine, phentermine, tramadol, citalopram, venlafaxine, atomoxetine, amitriptyline, protriptyline, mirtazapine (10 shown, remaining drugs shown in Supplementary Table S3)
ITGA2B	100	2	Abciximab, tirofiban
SERPINB2	100	2	Urokinase, tenecteplase
ATF3	100	1	Pseudoephedrine
LHCGR	99.8	7	Goserelin, menotropins, lutropin alfa, cetrorelix, chorionic gonadotropin (recombinant), buserelin, chorionic gonadotropin (human)
LTF	99.8	2	Nimesulide, parecoxib
MPO	99.8	4	Mesalazine, cefdinir, l-carnitine, melatonin
ELANE	99.8	3	Pegfilgrastim, alpha-1-proteinase inhibitor, filgrastim

We also analyzed the expression variability of ADME genes (Supplementary Fig. S6). There were 284 such genes in this analysis, including 177 enzymes, 92 transporters and 21 carriers. These sets were not disjoint, since some genes belonged to several ADME categories, or were additionally the target of a certain drug (Supplementary Fig. S7). Similarly to drug target genes, the 284 ADME genes were highly variable, as shown previously (Chhibber *et al.*, 2017; Yang *et al.*, 2013, Supplementary Fig. S6A). Interestingly, the LCV values of ADME genes were not correlated with RE of the respective drugs (Supplementary Fig. S6B), and there was no significant difference between the LCV values of ADME genes related to approved and withdrawn drugs (Supplementary Fig. S6C).

### 3.4 Minor gender differences in the variability of drug target genes

**Fig. 5.**
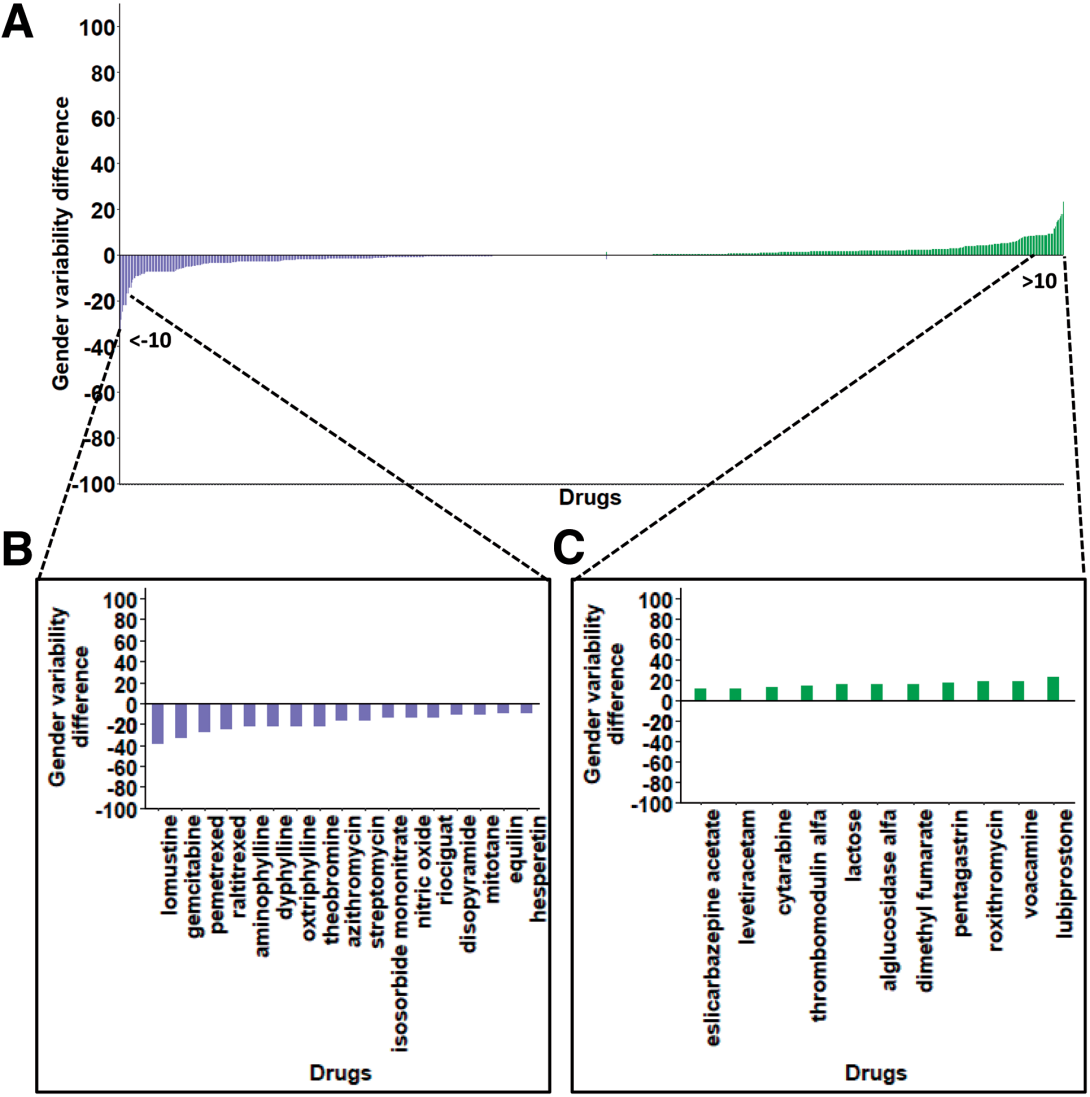
Minor gender differences observed for the most variable drug targets. **(A)** For each drug (of total 981 drugs), LCV values of its most variable target were computed separately for men and women, and the difference between them is shown (purple, higher LCV in women; green, higher LCV in men). Bar graphs show the drugs for which the LCV differences were above 10 between women **(B)** and men **(C)** (Color version of this figure is available at *Bioinformatics* online.)

Certain drugs elicit different responses between men and women (Franconi *et al.*, 2007). Here, we tested whether genes show a gender bias in terms of their expression variability. For this, we repeated the analyses described above upon considering only profiles of samples taken from either male or female subjects (see Section 2). The LCV values of drug target genes were highly similar between men and women (*r* = 0.96, Pearson correlation). In addition, the correlations we observed between RE and LCV were similar to those observed for all genes, and between men and women. Likewise, we obtained similar results upon comparing approved and withdrawn drugs (Supplementary Fig. S8). Nevertheless, the most variable targets of some drugs did have different LCVs (Fig. 5). For example, the gene GABRB2 is highly variable among women and less variable among men (LCV of 90 and 66.2, respectively). This gene is the target of several drugs, including propofol, midazolam and diazepam. Notably, a previous study showed that women are less sensitive (30–40%) than men to propofol, potentially due to having varying levels, and other differences were observed for midazolam and diazepam in women (Franconi *et al.*, 2007; Hoymork and Raeder, 2005). Another example involves ADORA1, which is the target of aminophylline. A sub-study of the ASSUGAE clinical trial reported that aminophylline treatment for the prevention of regadenoson-induced adverse events was more effective in men compared with women (Rangel *et al.*, 2013). Our analysis suggests that the higher variability of aminophylline targets in women (LCV of 86.2 compared with LCV of 64.2 in men) may contribute to its diminished effect.

## 4 Discussion

Here we examined the variable expression of human genes among individuals, particularly focusing on drug target genes. We hypothesized that the variability in the responses of patients to drugs could be related to differences in expression levels of drug target genes across individuals.

Our first step was to develop LCV as a robust measure for calculating variability. As we showed, LCV is completely unbiased by expression levels and assumes no a priori variability distribution. This makes LCV applicable to various types of experimental measurements without the need to tailor it to their variability distribution. Notably, LCV is related to other measures of variability that computed the variability of a gene relative to other genes with similar expression levels (Alemu *et al.*, 2014; Newman *et al.*, 2006; Silander *et al.*, 2012). Two of these studies used a running median, similar to our sliding window approach, and measured variability as the direct (DM, Newman *et al.*, 2006) or smoothed (Silander *et al.*, 2012) distance from this median. Another study assumed that variability was gamma-distributed over expression levels and computed variability as the ratio between the observed and expected gamma variability (EV, Alemu *et al.*, 2014). By measuring expression variability as the ranking of a gene amongst similarly expressed genes, as opposed to distance or ratio, LCV hides the absolute difference between expression variability values, and might inflate the variance of highly expressed genes. Nevertheless, ranking allows LCV to constitute a normalized measure that can be compared across different expression levels and across different datasets, and to remain completely unbiased by actual expression levels. LCV surpassed other measures when analyzing lowly expressed (usually not protein-coding) genes, where expression bias was the highest (Fig. 2C–G). When analyzing protein-coding genes, DM and EV generally showed similar results to LCV; however were less robust to incompleteness of data (Fig. 2H). Notably, unlike some of the other measures, LCV is a straightforward, easily computed measure that does not require extensive computational resources.

We applied LCV to assess the expression variability of 14 659 protein-coding genes in 19 different tissues, as measured via RNA-sequencing of tissue samples by GTEx (Consortium *et al.*, 2017). Previous analysis of these data showed that physiologically related human tissues have similar patterns of gene expression, implying that they have related regulatory programs (Mele *et al.*, 2015). Here, we found that physiologically related tissues also have similar expression variability patterns (Fig. 3A). This suggests that expression regulation and expression variability are intertwined. Highly variable genes and less variable genes were enriched for certain gene functions that correspond to a loose or tight regulation of expression, respectively. For example, receptors and environmental response pathways were enriched among highly variable genes, whereas nuclear components and pathways were enriched among less variable genes. These trends were consistent with previous analyses of the variability in protein levels in yeast (Newman *et al.*, 2006) and RNA levels in human (Alemu *et al.*, 2014), indicating that expression variability as measured by LCV reflects regulatory strategies that were conserved from yeast to human.

Upon analyzing the expression variability of 1176 drug target genes, we found them to be significantly more variable than protein-coding genes in general (Fig. 3B). Since drug targets tend to be involved in response to chemicals, and frequently function as receptors or membrane-bound proteins, their higher variability is expected given the trends that we and others observed among highly variable genes. We further hypothesized that the targeting of variable genes might lead to variable drug response among individuals. Indeed, our analysis of 1076 drugs showed that drugs that were found to be less effective in the general population tended to target highly variable genes (Fig. 4A and B). In accordance with this observation, in both glucocorticoids and Type III anti-arrhythmic agents, the drugs that targeted highly variable genes were less effective in the general population than other drugs of the same class. Furthermore, upon comparing withdrawn and approved drugs, we found that withdrawn drugs tend to target more variable genes than approved drugs (Fig. 4C and D). Although expression variability and drug responses were measured on different subjects, the large numbers of reports and samples and the statistical significance of our results support their validity.

Notably, there are some limitations to the datasets that were utilized in this study. To carry a cross-tissue analysis we relied on data from GTEx, which is the largest single resource of human tissue RNA-seq profiles to date. However, the usage of GTEx data to assess gene expression variability is problematic, since several parameters were shown to affect the expression levels of genes, such as post-mortem ischemic time, sequencing depth and RNA and sequencing quality (Consortium *et al.*, 2017), as well as distinct post-death transcriptional programs per tissue (Ferreira *et al.*, 2018). Thus, gene expression variability could stem from multiple sources, and could be distinct across tissues. Nevertheless, physiologically related tissues clustered together by the variability patterns of genes (Fig. 3A), and the properties of essential genes and genes with high or low variability were similar to those identified in studies based on other data (Alemu *et al.*, 2014) and in other organisms (Newman *et al.*, 2006), supporting our analyses. Another limitation relates to the drug effectiveness scores that we used (RE, Guney *et al.*, 2016). These scores were computed based on text-mining techniques that were applied to reports made to the FARES system by various entities, such as healthcare workers, patients and family members, or product manufacturers. These reports are subjective and often not medically verified, making FAERS a problematic database for estimating rates of adverse events or drug effectiveness in a population. However, testing for a relationship between drug effectiveness and variability required a large dataset of drug effectiveness scores computed systematically for a wide array of drugs. Despite the shortcomings of RE scores, they were less likely to affect, in a consistent manner, results based on large-scale data of drugs. Although the identified correlations were weak and potentially questionable due to multiple hypothesis testing, the trends we observed were repeated in separate analysis of data from men and women (Supplementary Fig. S8), partially repeated upon analyzing small subsets of drugs in disease segments (Supplementary Table S1), and consistent with the variability difference between approved and withdrawn drugs, hence strengthening their validity.

Although gender is known to play a role in the variability of drug response (Fisher and Ronald, 2010; Soldin and Mattison, 2009; Tamargo *et al.*, 2017), it was typically attributed to physiological differences between males and females that affect pharmacokinetic and pharmacodynamics factors. Some of these may be manifested by differences in the expression of ADME genes, yet here we observed only minor gender differences. Interestingly, while ADME genes tend to be even more variable than drug target genes, their variability is not similarly related to drug effectiveness. It is possible that these genes, responsible for the processing of drugs in the body, have more ‘responsive’ expression regulation mechanisms than most drug target genes. Therefore, they could be generally more robust to inter-individual baseline differences in expression.

The safety and effectiveness of drugs in the general population are typically uncovered with the introduction of a drug to widespread use. The mechanisms that govern drug effectiveness are extremely complex and involve many factors, such as genetic variations of pharmacogenes (Eichler *et al.*, 2011), and expression variability of ADME genes (Chhibber *et al.*, 2017; Yang *et al.*, 2013). Here we show for the first time that expression variability of drug target genes is related, at least in part, to drug effectiveness in the general population, and is particularly relevant for drugs that target highly variable genes. Given the availability of robust expression variability measures and rich data for their computation, we propose that expression variability of drug targets is worth considering upon drug treatment and development.

## Supplementary Material

btz023_Supplementary_Data
