# Supplementary material for: Large-scale analysis of human gene expression variability associates highly variable drug targets with lower drug effectiveness and safety
Source: Bioinformatics. 2019 Jan 14;35(17):3028–37. doi: 10.1093/bioinformatics/btz023 (PMC6735839; doi:10.1093/bioinformatics/btz023)
Supplement: btz023_Supplementary_Data [file btz023_supplementary_data.zip › btz023-suppl_data/btz023_SI_Nov24.docx]

**Large-scale analysis of human gene expression variability associates highly variable drug targets with lower drug effectiveness and safety: Supplementary Information**

Eyal Simonovsky^1^, Ronen Schuster^1^ and Esti Yeger-Lotem^1,2,*^

^1^Department of Clinical Biochemistry & Pharmacology, Ben-Gurion University of the Negev, Beer-Sheva 84105, Israel; ^2^National Institute for Biotechnology in the Negev, Ben-Gurion University of the Negev, Beer-Sheva 84105, Israel

- Corresponding author: [estiyl@bgu.ac.il](mailto:estiyl@bgu.ac.il)

This file contains the following Supplementary Figures and Tables:

Figure S1. The number of RNA-sequencing profiles available for each tissue in our dataset.

Figure S2. Spearman correlations between different expression variability measures and window sizes.

Figure S3. Variability distribution changes in LCV values by window sizes and expression levels for cerebellum profiles.

Figure S4. The correlations between LCV and gene expression levels computed for different window sizes.

Figure S5. Essentiality of protein-coding genes is negatively correlated with variability.

Figure S6. The expression variability of ADME genes.

Figure S7. Numbers of drug targets and ADME genes included in the analysis, and intersections.

Figure S8. The relationship between expression variability of drug targets and drug effectiveness is also observed in separate analyses of data from men and women.

Figure S9. RE score distribution for drug target genes.

Figure S10. Distribution of number of target genes per drug.

Table S1: The correlation between LCV and RE per drug class.

Table S2: The difference between approved and withdrawn drugs per drug class.

**Figure S1. The number of RNA-sequencing profiles available for each tissue in our dataset.**

We analyzed the expression variability of genes in the 19 tissues with 10 profiles or more, as marked by the horizontal line.


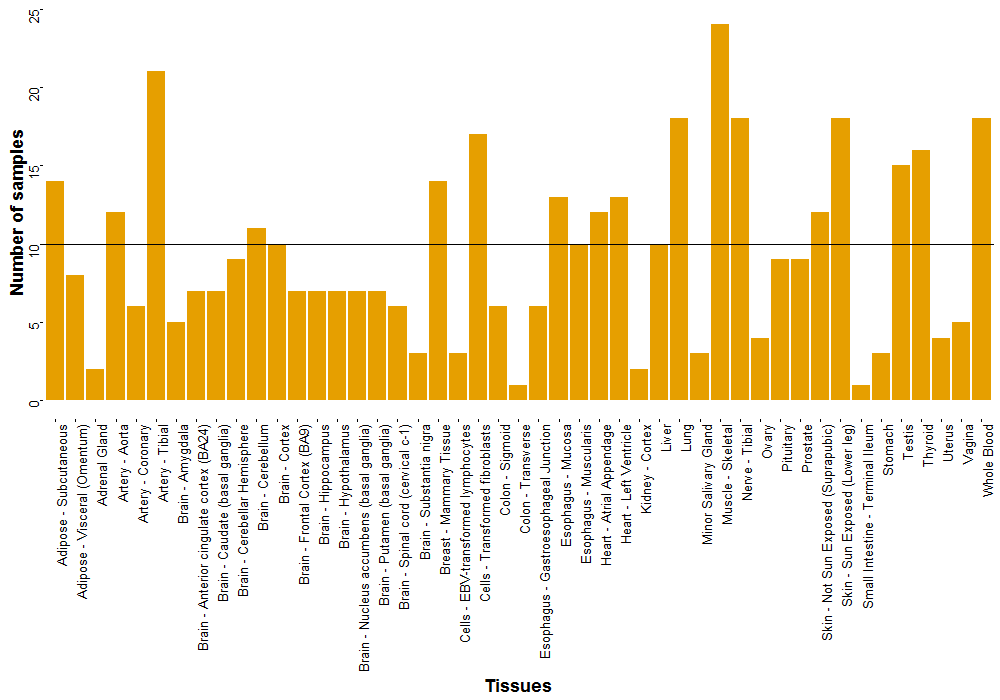


**Figure S2. Spearman correlations between different expression variability measures and window sizes.**

Each entry represents the correlation between the median variability of genes across human tissues, as measured by the two methods listed on the respective row and column. ‘LCV-number’ lists the window size used upon computing LCV. A color legend for correlation values appears on the right.


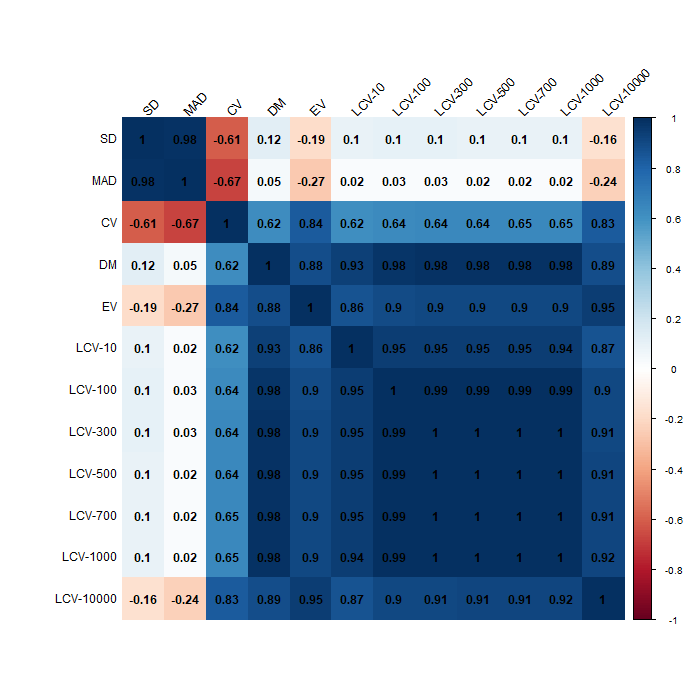


**Figure S3. Variability distribution changes in LCV values by window sizes and expression levels for cerebellum profiles.**

Red lines depicts LOWESS locally-weighted polynomial regression


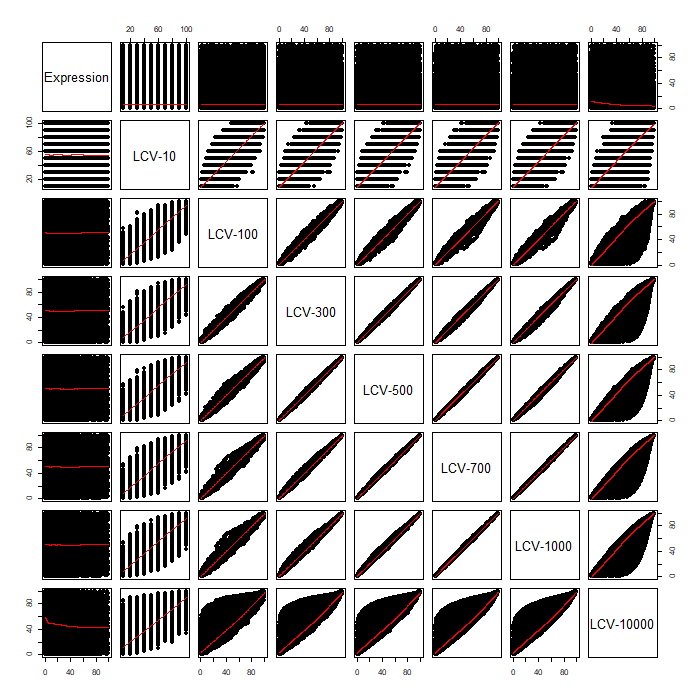


**Figure S4. The correlations between LCV and gene expression levels computed for different window sizes.**

Each dot represents the Spearman correlation between LCV and gene expression levels in a certain tissue (Y axis) for a specific window size (X axis). Red horizontal lines represent the median correlation across all 19 tested tissues.


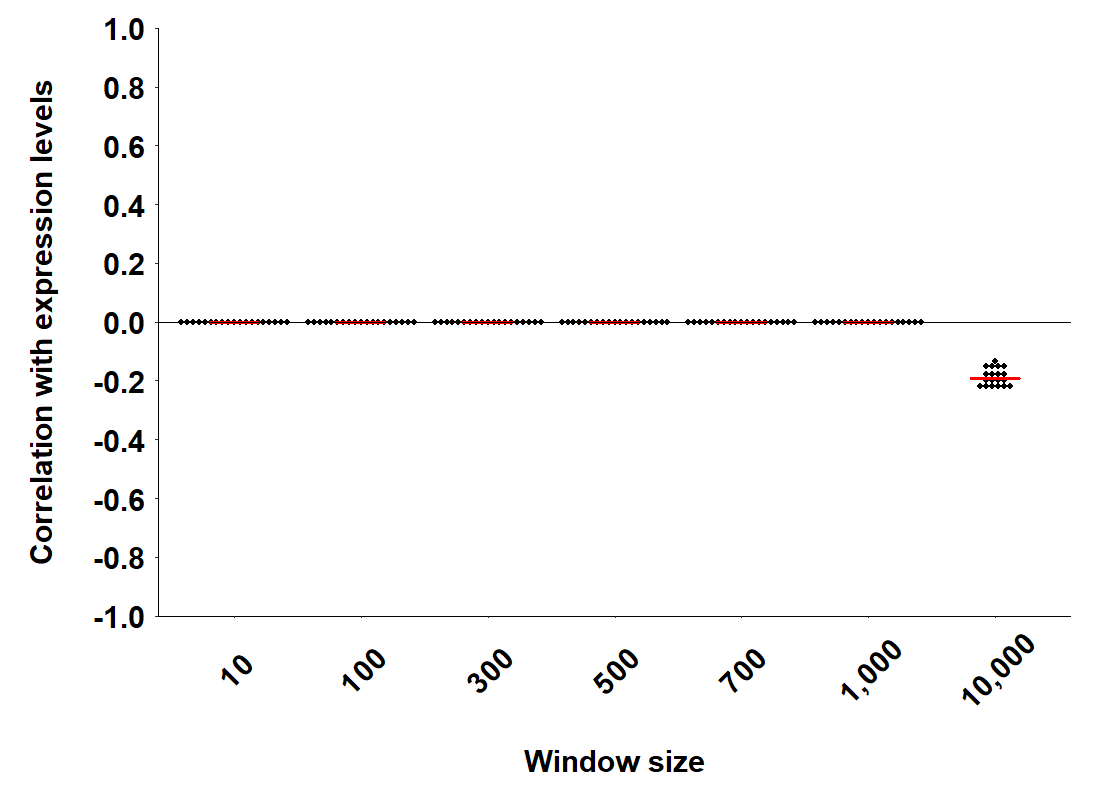


**Figure S5. Essentiality of protein-coding genes is negatively correlated with variability.**

The essentiality of 14,056 genes is plotted against their median expression variability (LCV) across tissues, showing that essential genes tended to be less variable (r=-0.25, p=1.5E-200 Spearman correlation).


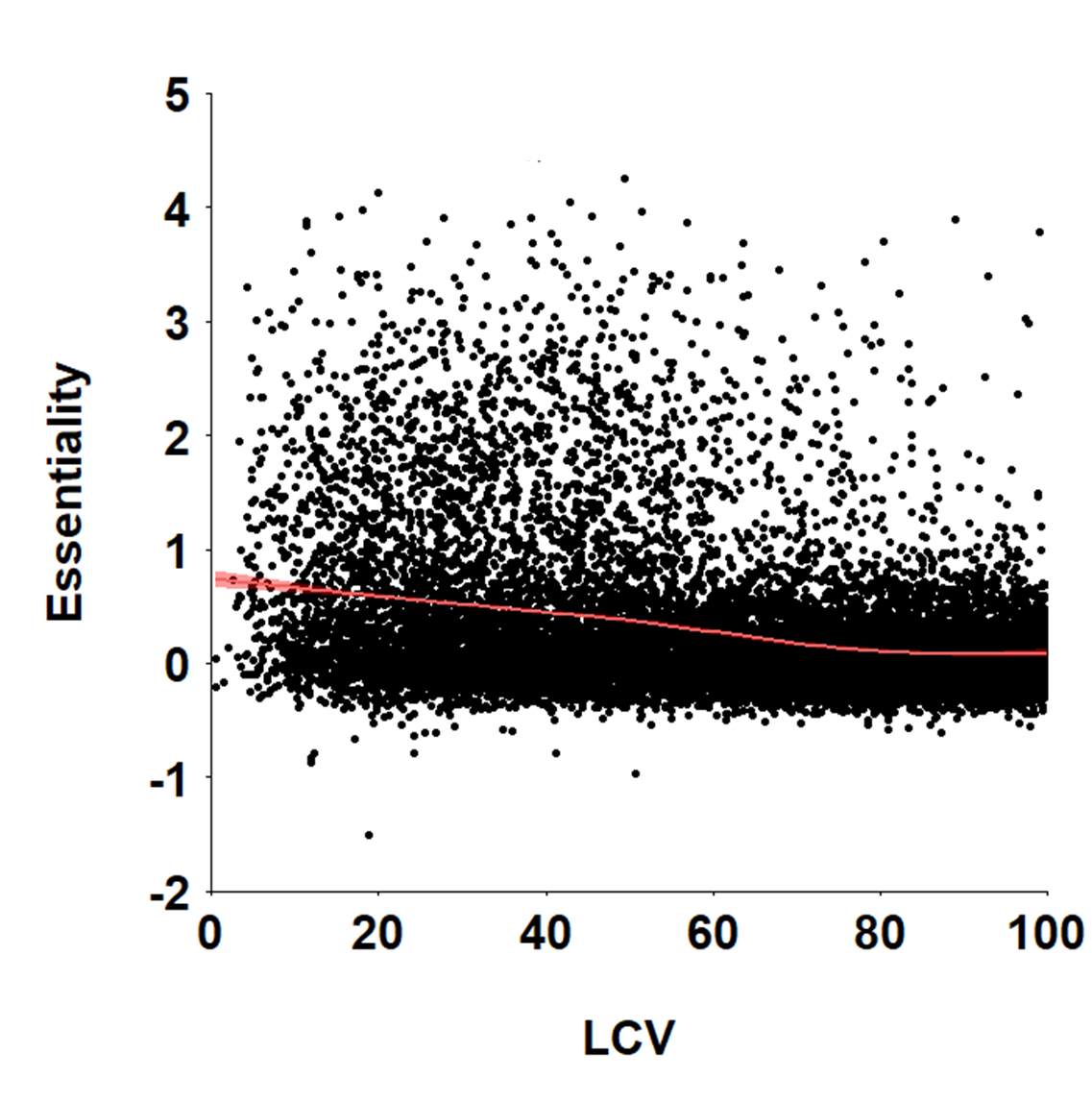


**Figure S6. The expression variability of ADME genes.**

1. The variability of ADME genes across tissues. The median LCV scores of ADME genes across tissues show that ADME genes are highly variable (left), and that this tendency is consistent per family (right, the median LCV of protein coding genes shows by the horizontal red line, classification to ADME gene families according to DrugBank).
2. The relative efficacy (RE) of drugs and the expression variability of their most variable ADME genes are not correlated (r=0.02, p=0.84, Spearman correlation).
3. The expression variability of ADME genes is shown for the set of approved drugs and for the set of drugs that were withdrawn from the market. Each dot represents the most variable ADME gene related to the drug. The expression variability of ADME genes that are related to withdrawn and approved drugs is similar (p=0.37 Mann-Whitney U tests).


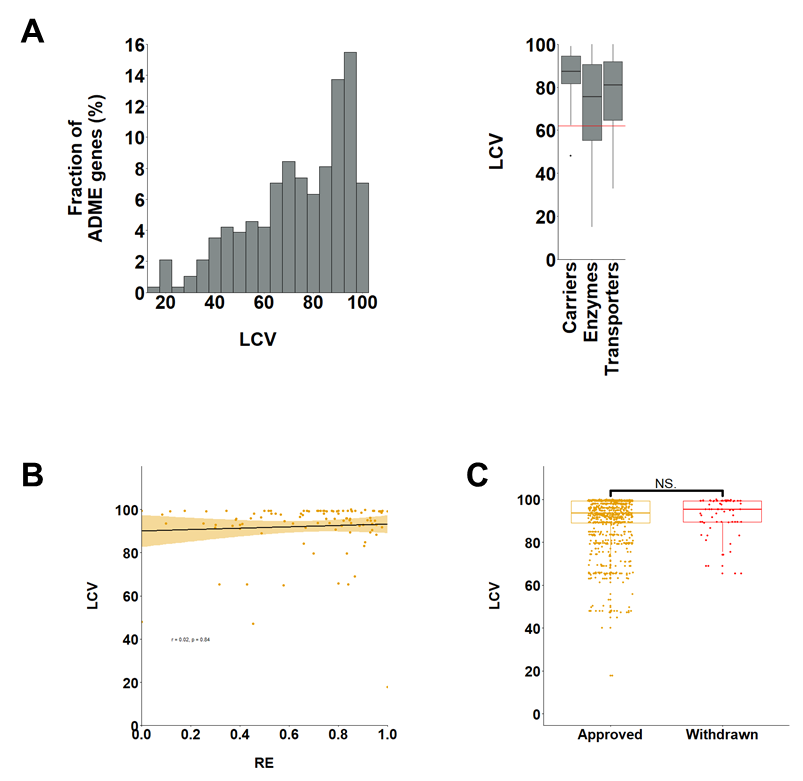


**Figure S7. Numbers of drug targets and ADME genes included in the analysis, and intersections.**

A. Venn diagram of drug targets and ADME genes.

B. Venn diagram of different ADME gene categories.


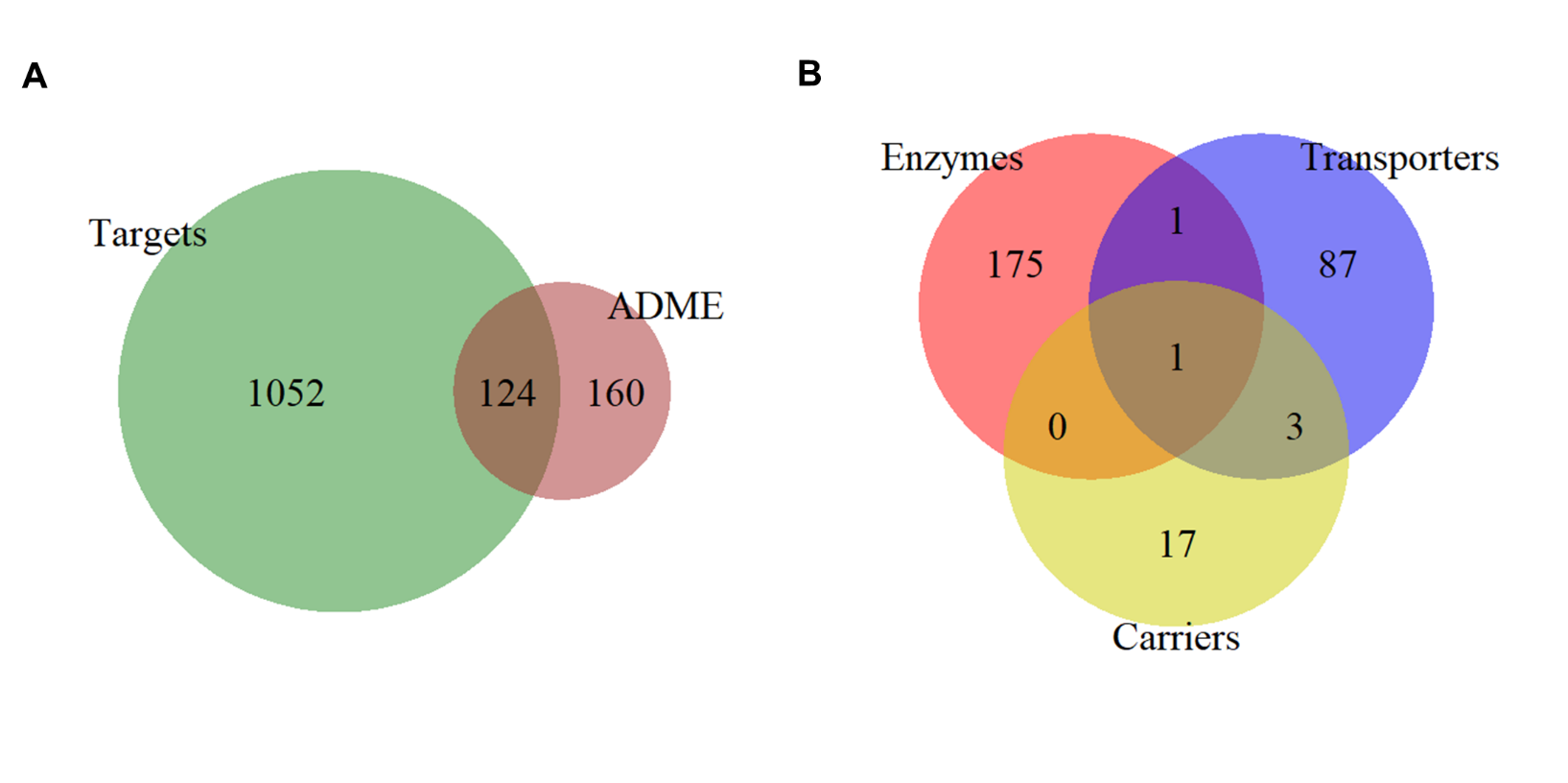


**Figure S8. The relationship between expression variability of drug targets and drug effectiveness is also observed in separate analyses of data from men and women.**

1. The relative efficacy (RE) of a drug is plotted against the expression variability (LCV) of its most variable target gene using the gender analysis subset of data for male and female (left, orange); only male (center, green); and only female (right, purple). In all cases, mild but significant negative correlations were obtained between RE and LCV
   (Spearman r=-0.32, -0.33, -0.27, respectively).
2. Expression variability of drug target genes is shown for the set of approved drugs and for the set of drugs that were withdrawn from the market, calculated using the gender analysis subset of data for male and female (left, orange); only male (center, green); and only female (right, purple). Each dot represents the most variable target of the drug. The targets of withdrawn drugs are significantly more variable than the targets of approved drugs (p<0.001 regardless of gender, Mann-Whitney U test).
3. Male- and female-based LCV values of drug target genes are highly correlated (r=0.96, p<1*10^-15^; Pearson correlation).


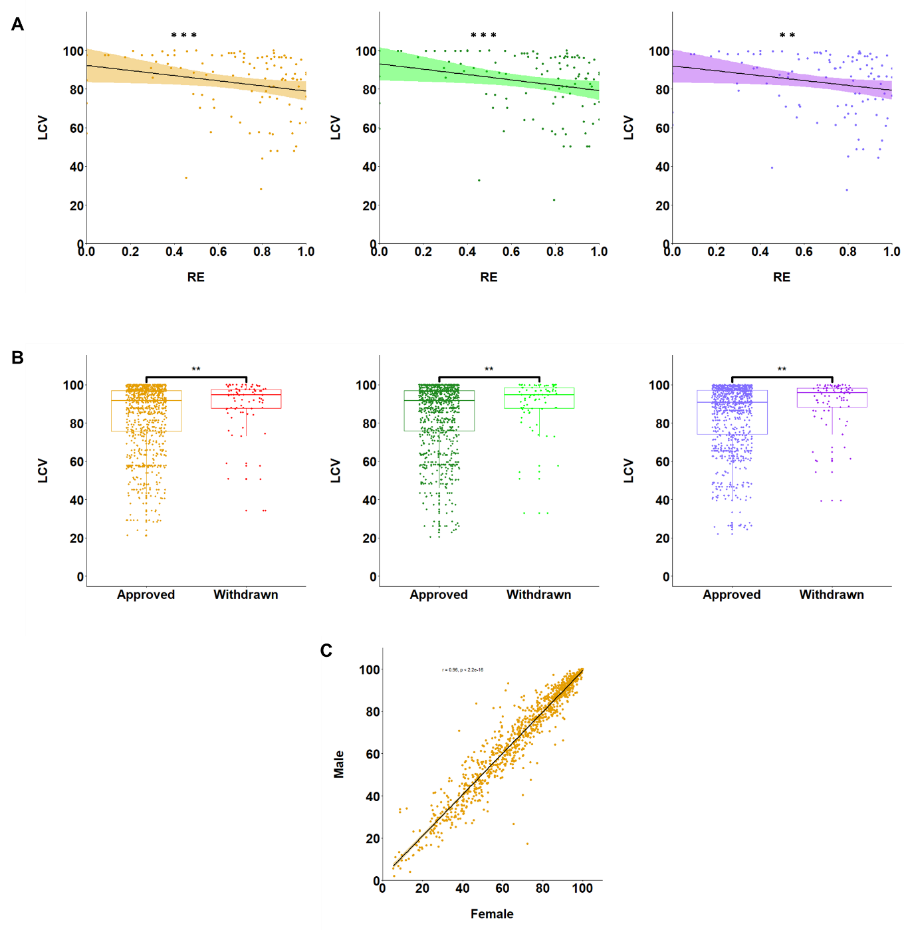


**Figure S9. RE score distribution for drug target genes.**


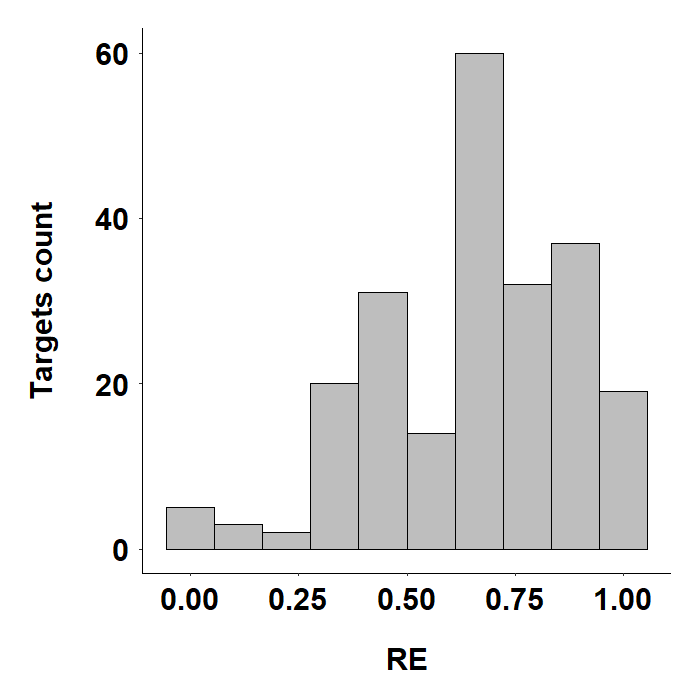


**Figure S10. Distribution of number of target genes per drug.**


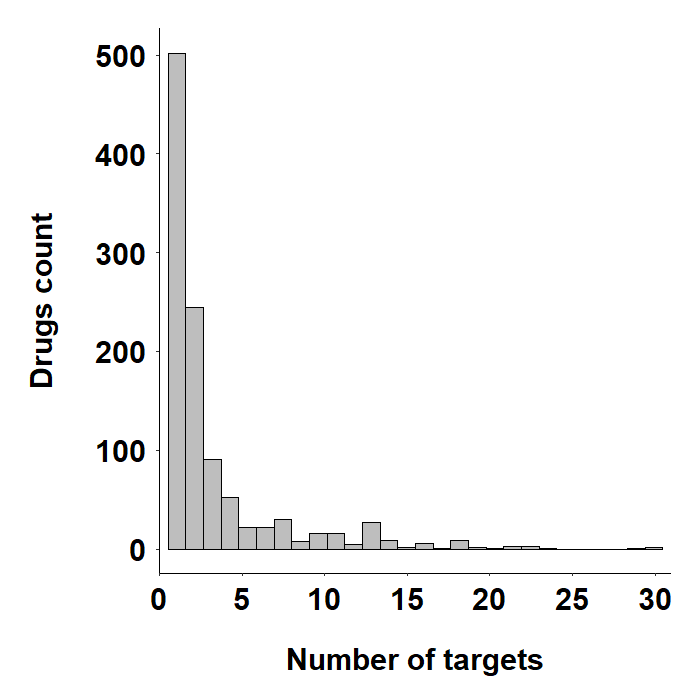


**Table S1: The correlation between LCV and RE per drug class.**

| ATC code* | Code name | # of drugs | r | p |
| --- | --- | --- | --- | --- |
| A | Alimentary tract and metabolism | 15 | -0.57 | 0.027 |
| C | Cardiovascular system | 18 | -0.35 | 0.16 |
| L | Antineoplastic and immunomodulating agents | 26 | -0.031 | 0.88 |
| M | Musculo-skeletal system | 10 | -0.72 | 0.019 |
| N | Nervous system | 17 | 0.33 | 0.2 |
| R | Respiratory system | 10 | -0.38 | 0.29 |

* We computed the correlation for groups consisting of at least 10 observations.

**Table S2: The difference between approved and withdrawn drugs per drug class.**

| ATC code* | Code name | # of approved drugs | # of withdrawn drugs | Median LCV of approved | Median LCV of withdraw | p |
| --- | --- | --- | --- | --- | --- | --- |
| A | Alimentary tract and metabolism | 99 | 10 | 84.8 | 93.8 | 0.005 |
| C | Cardiovascular system | 102 | 10 | 85.25 | 90.00 | 0.43 |
| N | Nervous system | 144 | 20 | 97.6 | 94.7 | 0.53 |

* We computed the difference for groups that contained at least 10 approved and 10 withdrawn drugs, resulting in only 3 groups. In one group (A), withdrawn drugs indeed targeted significantly more variable genes (p=0.005).
